# Briarenolides U–Y, New Anti-Inflammatory Briarane Diterpenoids from an Octocoral *Briareum* sp. (Briareidae)

**DOI:** 10.3390/md13127060

**Published:** 2015-12-03

**Authors:** Yin-Di Su, Tung-Ying Wu, Zhi-Hong Wen, Ching-Chyuan Su, Yu-Hsin Chen, Yu-Chia Chang, Yang-Chang Wu, Jyh-Horng Sheu, Ping-Jyun Sung

**Affiliations:** 1 Department of Marine Biotechnology & Resources and Asia-Pacific Ocean Research Center, National Sun Yat-sen University, Kaohsiung 804, Taiwan; gobetter04@yahoo.com.tw (Y.-D.S.); wzh@mail.nsysu.edu.tw (Z.-H.W.); 2 National Museum of Marine Biology & Aquarium, Pingtung 944, Taiwan; kb5634@yahoo.com.tw (Y.-H.C.); jay0404@gmail.com (Y.-C.C.); 3 Chinese Medicine Research and Development Center, China Medical University Hospital, Taichung 404, Taiwan; kuma0401@gmail.com; 4 Doctoral Degree Program of Marine Biotechnology, National Sun Yat-sen University & Academia Sinica, Kaohsiung 804, Taiwan; 5 Antai Medical Care Cooperation Antai Tian-Sheng Memorial Hospital, Pingtung 928, Taiwan; a081001@mail.tsmh.org.tw; 6 Department of Beauty Science, Meiho University, Pingtung 912, Taiwan; 7 Department of Life Science and Institute of Biotechnology, National Dong Hwa University, Hualien 974, Taiwan; 8 School of Pharmacy, College of Pharmacy, China Medical University, Taichung 404, Taiwan; 9 Center for Molecular Medicine, China Medical University Hospital, Taichung 404, Taiwan; 10 Graduate Institute of Natural Products, Kaohsiung Medical University, Kaohsiung 807, Taiwan; 11 Graduate Institute of Marine Biology, National Dong Hwa University, Pingtung 944, Taiwan

**Keywords:** *Briareum*, briarane, octocoral, anti-inflammatory, iNOS, COX-2

## Abstract

Five new 13,14-epoxybriarane diterpenoids, briarenolides U–Y (**1**–**5**), were isolated from the octocoral *Briareum* sp. The structures of briaranes **1**–**5** were elucidated by spectroscopic methods. Briarenolides U–Y (**1**–**5**) were found to significantly inhibit the expression of the pro-inflammatory inducible nitric oxide synthase (iNOS) and cyclooxygenase-2 (COX-2) protein of the lipopolysaccharide (LPS)-stimulated RAW264.7 macrophage cells.

## 1. Introduction

**Figure 1:**
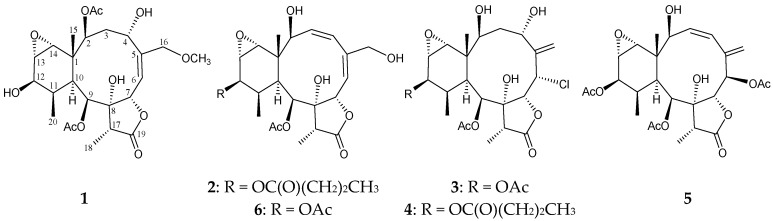
The structures of briarenolides U–Y (**1**–**5**) and briaexcavatolide N (**6**).

Since the isolation in 1977 of the first briarane-type natural product from the Caribbean gorgonian *Briareum asbestinum* [1], hundreds of the compounds of this type were obtained from various marine organisms and mainly from octocorals belonging to the genus *Briareum* [2,3,4,5,6]. Previous studies on the chemical constituents of *Briareum* spp. collected off the waters of Taiwan, have yielded a series of briarane metabolites [2,3,4,5,6]. In our continuing studies of this interesting organism, a sample collected at the Southern Tip, Taiwan, identified as *Briareum* sp., yielded five new briaranes, briarenolides U–Y (**1**–**5**) (Figure 1). In this paper, we report the isolation, structure determination, and anti-inflammatory activity of briaranes **1**–**5**.

## 2. Results and Discussion

Briarenolide U (**1**) was isolated as a white powder. The molecular formula of **1** was established as C_25_H_36_O_11_ (eight degrees of unsaturation) from a sodium adduct at *m*/*z* 535 in the electrospray ionization mass spectrum (ESIMS) and further supported by the high-resolution electrospray ionization mass spectrum (HRESIMS) at *m*/*z* 535.21480 (calcd. for C_25_H_36_O_11_ + Na, 535.21498). The IR spectrum of **1** showed bands at 3445, 1770 and 1733 cm^−1^, consistent with the presence of hydroxy, γ-lactone and ester carbonyl groups. The ^13^C NMR and distortionless enhancement polarization transfer (DEPT) spectroscopic data showed that this compound has 25 carbons (Table 1), including six methyls, two sp^3^ methylenes, ten sp^3^ methines, two sp^3^ quaternary carbons, one sp^2^ methine and four sp^2^ quaternary carbons. From ^1^H and ^13^C NMR spectra (Table 1), **1** was found to possess two acetoxy groups (δ_H_ 2.23, 2.10, each 3H × s; δ_C_ 21.9, 21.3, 2 × CH_3_; 169.0, 172.6, 2 × acetate carbonyls), one γ-lactone moiety (δ_C_ 176.1, C-19) and a trisubstituted olefin (δ_H_ 5.66, 1H, dd, *J* = 10.4, 1.6 Hz, H-6; δ_C_ 147.5, C-5; 116.7, CH-6). The presence of one disubstituted epoxy group was established from the signals of two oxymethines at δ_C_ 63.1 (CH-14) and 59.1 (CH-13) and further confirmed by the proton signals at δ_H_ 2.92 (1H, d, *J* = 3.6 Hz, H-14) and 3.15 (1H, d, *J* = 3.6 Hz, H-13). On the basis of the above unsaturation data, **1** was concluded to be a diterpenoid molecule possessing four rings.

**Table 1:** ^1^H (400 MHz, CDCl_3_) and ^13^C (100 MHz, CDCl_3_) NMR data and ^1^H–^1^H COSY and HMBC correlations for briarane **1**.

Position	δ_H_ (*J* in Hz)	δ_C_, Multiple	^1^H–^1^H COSY	HMBC
1		41.2, C		
2	4.70 ddd (2.4, 2.0, 2.0)	77.6, CH	H_2_-3	C-1, -4, -10, -15, acetate carbonyl
3	3.11 ddd (15.2, 4.8, 2.0); 1.94 m	40.2, CH_2_	H-2, H-4	C-1, -2, -4, -5
4	4.85 br s	68.6, CH	H_2_-3, OH-4	n. o. ^a^
5		147.5, C		
6	5.66 dd (10.4, 1.6)	116.7, CH	H-7, H_2_-16	C-4, -16
7	5.02 d (10.4)	75.9, CH	H-6	C-5, -6
8		82.2, C		
9	5.22 d (5.2)	71.5, CH	H-10	C-7, -8, -10, -11, acetate carbonyl
10	1.81 dd (5.2, 2.8)	37.3, CH	H-9, H-11	C-1, -2, -8, -9, -11, -15, -20
11	1.97 m	42.2, CH	H-10, H-12, H_3_-20	n. o.
12	3.71 d (4.4)	70.2, CH	H-11	C-13, -20
13	3.15 d (3.6)	59.1, CH	H-14	C-1
14	2.92 d (3.6)	63.1, CH	H-13	C-1, -10, -13, -15
15	1.21 s	16.9, CH_3_		C-1, -2, -10, -14
16	4.36 br s	73.2, CH_2_	H-6	C-4, -5, -6, methoxy carbon
17	2.36 q (7.2)	42.5, CH	H_3_-18	C-8, -18, -19
18	1.16 d (7.2)	6.5, CH_3_	H-17	C-8, -17, -19
19		176.1, C		
20	1.07 d (7.2)	8.7, CH_3_	H-11	C-10, -11, -12
2-OAc		172.6, C		
	2.10 s	21.3, CH_3_		Acetate carbonyl
9-OAc		169.0, C		
	2.23 s	21.9, CH_3_		Acetate carbonyl
16-OCH_3_	3.46 s	58.9, CH_3_		C-16
OH-4	3.99 d (10.4)		H-4	n. o.

^a^ n. o. = not observed.

From the ^1^H–^1^H correlation spectroscopy (COSY) spectrum of **1** (Table 1), it was possible to establish the separate system that maps out the proton sequences from H-2/H_2_-3/H-4, H-6/H-7 and H-9/H-10. These data, together with the heteronuclear multiple-bond coherence (HMBC) correlations between H-2/C-1, -4, -10; H_2_-3/C-1, -2, -4, -5; H-6/C-4; H-7/C-5, -6; H-9/C-7, -8, -10; and H-10/C-1, -2, -8, -9, established the connectivity from C-1 to C-10 in the 10-membered ring (Table 1). The methylcyclohexane ring, which is fused to the 10-membered ring at C-1 and C-10, was elucidated by the ^1^H–^1^H COSY correlations between H-10/H-11/H-12, H-13/H-14, and H-11/H_3_-20 and by the HMBC correlations between H-9/C-11; H-10/C-11, -20; H-12/C-13, -20; H-13/C-1; H-14/C-1, -10, -13 and H_3_-20/C-10, -11, -12. The ring junction C-15 methyl group was positioned at C-1 from the HMBC correlations between H_3_-15/C-1, -2, -10, -14 and H-2, H-10, H-14/C-15. The acetate esters at C-2 and C-9 were established by the correlations between H-2 (δ_H_ 4.70), H-9 (δ_H_ 5.22) and the acetate carbonyls at δ_C_ 172.6 and 169.0, respectively, in the HMBC spectrum of **1**. The methoxy group at C-16 was confirmed by the HMBC correlations between the oxymethylene protons at δ_H_ 4.36 (H_2_-16) and C-4 (δ_C_ 68.6), -5 (δ_C_ 147.5), -6 (δ_C_ 116.7) and an oxygenated methyl carbon at δ_C_ 58.9, and further confirmed by the allylic couplings between H_2_-16 and H-6. The presence of a hydroxy group at C-4 was deduced from the ^1^H–^1^H COSY correlation between a hydroxy proton (δ_H_ 3.99) and H-4 (δ_H_ 4.85). Thus, the remaining hydroxy groups had to be attached at C-8 and C-12 positions, respectively. These data, together with the ^1^H–^1^H COSY correlation between H-17 and H_3_-18 and the HMBC correlations between H-17/C-8, -18, -19 and H_3_-18/C-8, -17, -19, were used to establish the molecular framework of **1**.

**Figure 2:**
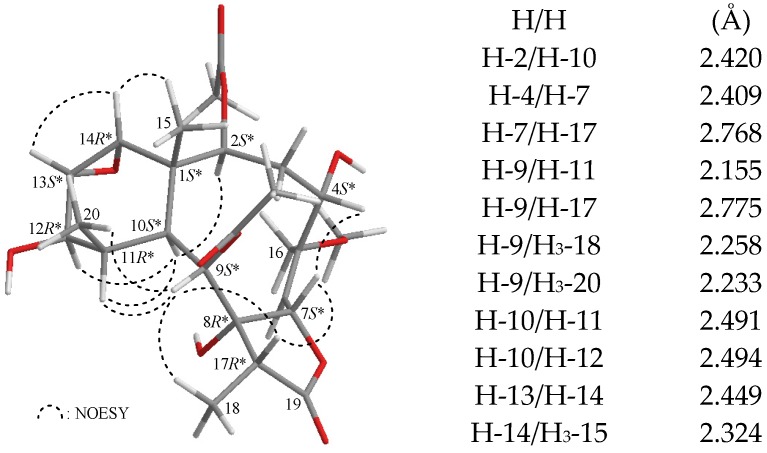
The computer-generated model of **1** using MM2 force field calculations and the calculated distances (Å) between selected protons with key NOESY correlations.

In all naturally-occurring briarane-type natural products, H-10 is *trans* to the C-15 methyl group at C-1, and these two groups are assigned as α- and β-oriented, respectively, in briarane derivatives. The relative configuration of **1** was elucidated from the interactions observed in a nuclear Overhauser effect spectroscopy (NOESY) experiment and was found to be compatible with that of **1** offered by computer modeling (Figure 2) [7]. In the NOESY experiment of **1**, the correlations of H-10 with H-2, H-11 and H-12, but not with H_3_-15 and H_3_-20, indicated that H-2, H-10, H-11, and H-12 were situated on the same face and were assigned as α protons, since the Me-15 and Me-20 are β-substituents at C-1 and C-11, respectively. H-14 showed correlations with H-13 and Me-15, but not with H-10, as well as a lack of coupling was detected between H-12 and H-13, indicating that the dihedral angle between H-12 and H-13 is approximately 90° and the 13,14-epoxy group has an α-orientation. H-9 was found to show responses to H-11, H-17, H_3_-18, and H_3_-20. From modeling analysis, H-9 was found to be close to H-11, H-17, H_3_-18, and H_3_-20 when H-9 was α-oriented. H-7 correlated with H-17, but not with H_3_-18, indicating that H-7 and 8-hydroxy group were β- and α-oriented, respectively, in the γ-lactone moiety. Furthermore, H-4 correlated with H-7, but not with H-2, confirming the β-orientation for this proton. From the above evidence, the relative configuration of chiral carbons of **1** was assumed to be 1S*, 2S*, 4S*, 7S*, 8R*, 9S*, 10S*, 11R*, 12R*, 13S*, 14R*, and 17R*. Based on the above findings, the structure, including the relative configuration of **1**, was fully determined.

Briarenolide V (**2**) was isolated as a white powder and had a molecular formula of C_26_H_36_O_10_ on the basis of HRESIMS at *m*/*z* 531.22025 (C_26_H_36_O_10_ + Na, calcd. 531.22007). Carbonyl resonances in the ^13^C NMR spectrum of **2** (Table 2) at δ_C_ 177.2, 173.6 and 170.1 revealed the presence of a γ-lactone and two other esters in **2**. In the ^1^H NMR spectrum of **2** (Table 2), a signal for one acetate methyl group was observed at δ_H_ 2.18 (3H, s). The additional acyl group was found to be an *n*-butyrate group, which showed seven contiguous protons (δ_H_ 0.96, 3H, t, *J* = 7.2 Hz; 1.66, 2H, sext, *J* = 7.2 Hz; 2.35, 2H, t, *J* = 7.2 Hz). The ^13^C NMR signal at δ_C_ 173.6 correlated with the methylene protons at δ_H_ 2.35 in the HMBC spectrum and was consequently assigned as the carbon atom of the *n*-butyrate carbonyl.

**Table 2:** ^1^H (400 MHz, CDCl_3_) and ^13^C (100 MHz, CDCl_3_) NMR data and ^1^H–^1^H COSY and HMBC correlations for briarane **2**.

Position	δ_H_ (*J* in Hz)	δ_C_, Multiple	^1^H–^1^H COSY	HMBC
1		40.4, C		
2	4.11 d (10.4)	75.9, CH	H-3	C-1, -4, -15
3	5.80 dd (10.4, 10.4)	135.9, CH	H-2, H-4	C-5
4	6.31 d (10.4)	125.5, CH	H-3	n. o. ^a^
5		145.3, C		
6	5.72 d (8.8)	120.0, CH	H-7	C-4, -16
7	5.23 d (8.8)	79.9, CH	H-6	n. o.
8		81.6, C		
9	5.15 d (6.8)	70.1, CH	H-10	C-8, -10, -11, -17, acetate carbonyl
10	1.96 m	37.4, CH	H-9, H-11	C-1, -2, -8, -9, -11, -15, -20
11	2.07 m	37.8, CH	H-10, H-12, H_3_-20	C-10
12	4.73 d (4.4)	71.8, CH	H-11	C-13, C-1′
13	3.18 br s	57.8, CH		C-1
14	3.18 br s	62.8, CH		C-1, -15
15	1.13 s	15.1, CH_3_		C-1, -2, -10, -14
16	4.29 br s	63.6, CH_2_		n. o.
17	2.28 q (7.2)	43.4, CH	H_3_-18	C-8, -18, -19
18	1.15 d (7.2)	6.4, CH_3_	H-17	C-8, -17, -19
19		177.2, C		
20	1.04 d (7.2)	9.5, CH_3_	H-11	C-10, -11, -12
9-OAc		170.1, C		
	2.18 s	21.8, CH_3_		Acetate carbonyl
12-OC(O)CH_2_CH_2_CH_3_				
1′ 2′ 3′ 4′				
1′		173.6, C		
2′	2.35 t (7.2)	36.2, CH_2_	H_2_-3′	C-1′, -3′, -4′
3′	1.66 sext (7.2)	18.3, CH_2_	H_2_-2′, H_3_-4′	C-1′, -2′, -4′
4′	0.96 t (7.2)	13.7, CH_3_	H_2_-3′	C-2′, -3′

^a^ n. o. = not observed.

It was found that the NMR signals (^1^H and ^13^C) of **2** were similar to those of a known briarane analogue, briaexcavatolide N (**6**) [8], except that the signals corresponding to an acetate group in **6** were replaced by signals for an *n*-butyrate group in **2**. The *n*-butyrate ester was positioned at C-12 from an HMBC correlation between H-12 (δ_H_ 4.73) and the carbonyl carbon of the *n*-butyrate (δ_C_ 173.6, C-1′) (Table 2). The correlations from a NOESY experiment of **2** also showed that the stereochemistry of this metabolite is identical with that of **6** and the relative configuration of chiral carbons of **2** were assumed to be 1S*, 2S*, 7S*, 8R*, 9S*, 10S*, 11R*, 12R*, 13S*, 14R*, and 17R*. Thus, briarenolide V (**2**) was found to be the 12-*O*-deacetyl-12-*O*-*n*-butyryl derivative of **6**.

Briarenolide W (**3**) had a molecular formula of C_24_H_3__3_ClO_1__0_ as derived from a quasi-molecular ion at *m*/*z* 539 [M + Na]^+^ in the ESIMS and from DEPT and ^13^C NMR spectra. Its IR bands indicated the presence of hydroxy (3461 cm^−^^1^), γ-lactone (1778 cm^−^^1^) and ester (1732 cm^−^^1^) groups. The ^1^H NMR data of **3** (Table 3) showed two acetyl singlets (δ_H_ 2.20, 2.12, each 3H × s), two methyl doublets (δ_H_ 1.14, 3H, d, *J* = 7.2 Hz, H_3_-18; 1.06, 3H, d, *J* = 6.8 Hz, H_3_-20) and a methyl singlet (δ_H_ 1.15, 3H, s, H_3_-15), an exocyclic carbon-carbon double bond (δ_H_ 6.03, 2H, br s, H_2_-16), three aliphatic methines (δ_H_ 1.95, 1H, m, H-10; 1.94, 1H, m, H-11; 2.38, 1H, q, *J* = 7.2 Hz, H-17), one aliphatic methylene (δ_H_ 2.46, 1H, br d, *J* = 16.4 Hz; 2.10, 1H, m, H_2_-3), one chloromethine (δ_H_ 5.07, 1H, br s, H-6), seven oxymethines (δ_H_ 4.21, 1H, br s, H-2; 4.54, 2H, d, *J* = 4.0 Hz, H-4 and H-12; 5.05, 1H, br s, H-7; 5.12, 1H, d, *J* = 5.2 Hz, H-9; 3.21, 1H, d, *J* = 3.2 Hz, H-13; 3.10, 1H, d, *J* = 3.2 Hz, H-14) and one hydroxy proton (δ_H_ 3. 37, 1H, s, OH-8).

**Table 3:** ^1^H (400 MHz, CDCl_3_) and ^13^C (100 MHz, CDCl_3_) NMR data and ^1^H–^1^H COSY and HMBC correlations for briarane **3**.

Position	δ_H_ (*J* in Hz)	δ_C_, Multiple	^1^H–^1^H COSY	HMBC
1		40.4, C		
2	4.21 br s	71.1, CH	H_2_-3	C-1
3	2.46 br d (16.4); 2.10 m	35.8, CH_2_	H-2, H-4	C-1
4	4.54 d (4.0)	70.9, CH	H_2_-3, H_2_-16	C-2, -5, -16
5		143.6, C		
6	5.07 br s	64.1, CH	H-7, H_2_-16	n. o. ^a^
7	5.05 br s	77.5, CH	H-6	n. o.
8		83.9, C		
9	5.12 d (5.2)	73.0, CH	H-10	C-1, -7, -8, -10, -11, -17, acetate carbonyl
10	1.95 m	37.4, CH	H-9, H-11	C-8
11	1.94 m	39.5, CH	H-10, H-12, H_3_-20	C-9
12	4.54 d (4.0)	72.8, CH	H-11, H-13	C-10, -13, -20, acetate carbonyl
13	3.21 d (3.2)	57.5, CH	H-12, H-14	n. o.
14	3.10 d (3.2)	63.3, CH	H-13	C-1
15	1.15 s	16.7, CH_3_		C-1, -2, -10, -14
16	6.03 br s	120.6, CH_2_	H-4, H-6	C-4, -5, -6
17	2.38 q (7.2)	44.0, CH	H_3_-18	C-8, -9, -18, -19
18	1.14 d (7.2)	7.1, CH_3_	H-17	C-8, -17, -19
19		174.7, C		
20	1.06 d (6.8)	9.5, CH_3_	H-11	C-10, -11, -12
9-OAc		169.5, C		
	2.20 s	21.9, CH_3_		Acetate carbonyl
12-OAc		170.2, C		
	2.12 s	21.0, CH_3_		Acetate carbonyl
OH-8	3.37 s			C-7, -8, -9

^a^ n. o. = not observed.

The planar structure of **3** was determined by 2D NMR studies. The ^1^H–^1^H COSY experiment of **3** established the following correlations: H-2/H_2_-3/H-4, H-6/H-7, H-9/H-10/H-11/H-12/H-13/H-14, H-17/H_3_-18 and H-11/H_3_-20 (Table 3). These observations together with the HMBC correlations between H-2/C-1; H_2_-3/C-1; H-4/ C-2, -5; H-9/C-1, -7, -8, -10, -11; H-10/C-8; H-11/C-9; H-12/C-10, -13; H-14/C-1; and OH-8/C-7, -8, -9, established the connectivity from C-1 to C-14 (Table 3). The exocyclic carbon-carbon double bond at C-5 was elucidated by the HMBC correlations between H-4/C-16 and H_2_-16/C-4, -5, -6, and further confirmed by the allylic couplings between H-4/H_2_-16 and H-6/H_2_-16. The intensity of [M + Na + 2] isotope peak observed in the ESIMS [(M + Na)/(M + 2 + Na) = 3:1] was strong evidence of the presence of a chlorine atom in **3**. Consequently, the methine proton signal at δ_H_ 5.07 (1H, br s) was confidently assigned to H-6, which beared a chlorinated carbon (δ_C_ 64.1, CH-6), and was confirmed by the ^1^H–^1^H COSY correlations between H-6/7 and H-6/16 (by allylic coupling); and by the HMBC correlations between H_2_-16/C-4, -5, -6. C-15 methyl group was positioned at C-1 from the HMBC correlations between H_3_-15/C-1, -2, -10, -14. Furthermore, seven oxymethine protons were observed at δ_H_ 5.12, 5.05, 4.54, 4.54, 4.21, 3.21, 3.10, were ^1^*J*-correlated to the carbons δ_C_ 73.0, 77.5, 72.8, 70.9, 71.1, 57.5, 63.3, and assigned to C-9, -7, -12, -4, -2, -13, -14, respectively. In addition, the presence of two acetate esters at C-9 and C-12 was established by the correlations between H-9 (δ_H_ 5.12), H-12 (δ_H_ 4.54) and the acetate carbonyls at δ_C_ 169.5 and 170.2, respectively, observed in the HMBC spectrum of **3**. The relative stereochemistry of **3** was elucidated from the NOE interactions observed in a NOESY experiment. Due to the α-orientation of H-10, the ring junction C-15 methyl group should be β-oriented as no correlation was observed between H-10 and H_3_-15. The correlations between H-14/H_3_-15 and H-13/H-14, indicated the β-orientations of H-13 and H-14. In addition, the NOE correlations between H-10/H-2, OH-8, H-9, H-11, H-12, H_3_-18, suggested the α-orientation of these protons (H-2, H-9, H-10, OH-8, H-11, H-12 and H_3_-18) and H-17 is β-oriented. Furthermore, H-7 showed correlations with H-17 and H-6, suggesting that these protons are on the β face of **3**. Based on the above findings, the configurations of all chiral centers of **3** were assigned as 1S*, 2S*, 4S*, 6S*, 7R*, 8R*, 9S*, 10S*, 11R*, 12R*, 13S*, 14R*, and 17R*.

Briarenolide X (**4**), C_26_H_37_ClO_10_ (HRESIMS, *m*/*z* 567.19687, calcd. for C_26_H_37_ClO_10_ + Na, 567.19675), was recognized as a 6-chlorinated briarane diterpenoid closely related to **3** from their NMR data (Table 3 and Table 4). Both briaranes **3** and **4** have identical substituents: secondary hydroxy groups at C-2 and C-4; an exocyclic methylene at C-5; a chloride atom at C-6; a tertiary hydroxy group at C-8; a secondary acetate at C-9. They also have the C-13/14 epoxy group in common. While briarane **3** showed the presence of a secondary acetate at C-12 of the methylcyclohexane ring, **4** showed an *n*-butyrate at this position. The ^1^H and ^13^C NMR data assignments of briarenolide X (**4**) were made in comparison with the values of **3**. The position of the *n*-butyrate group at C-12 was corroborated by an HMBC correlation observed between *n*-butyrate carbonyl carbon at δ_C_ 172.7 and the proton at δ_H_ 4.58 (H-12) (Table 4). The other HMBC correlations observed fully supported the location of functional groups, and hence briarenolide X (**4**) was assigned as the structure **4** with the same relative stereochemistry as in briarane **3** because for the chiral carbons that **4** has in common with **3**, the ^1^H and ^13^C NMR chemical shifts and proton coupling constants matched well. Based on the above findings, the chiral carbons of **4** were assigned as 1S*, 2S*, 4S*, 6S*, 7R*, 8R*, 9S*, 10S*, 11R*, 12R*, 13S*, 14R*, and 17R*.

Briarenolide Y (**5**) was obtained as a white powder and the molecular formula for **5** was determined to be C_26_H_3__4_O_11_ (10 degrees of unsaturation) was confirmed by HRESIMS at *m*/*z* 545.19918 (calcd. for C_26_H_3__4_O_11_ + Na, 545.19933). Comparison of the ^1^H and DEPT spectra with the molecular formula indicated that there must be two exchangeable protons, requiring the presence of two hydroxy groups. The IR spectrum showed bands at 3445, 1770, and 1732 cm^−^^1^, consistent with the presence of hydroxy, γ-lactone and ester groups. From the ^13^C NMR data of **5** (Table 5), the presence of one disubstituted olefin and one exocyclic olefin were deduced from the signals at δ_C_ 138.1 (C-5), 134.7 (CH-3), 126.0 (CH-4), 122.2 (CH_2_-16) and further supported by four olefin proton signals at δ_H_ 6.07 (1H, d, *J* = 12.0 Hz, H-4), 5.80 (1H, dd, *J* = 12.0, 9.2 Hz, H-3), 5.63 (1H, s, H-16), and 5.50 (1H, s, H-16) in the ^1^H NMR spectrum of **5** (Table 5). Four carbonyl resonances appeared at δ_C_ 175.1, 170.6, 170.4, and 170.1 confirming the presence of a γ-lactone and three ester groups in **5**; three acetate methyls (δ_H_ 2.18, 2.13 and 2.09, each 3H × s) were also observed. So from the NMR data, six degrees of unsaturation were accounted for, and therefore **5** must be tetracyclic. The presence of one epoxide was elucidated from the signals of two oxymethines at δ_C_ 62.6 (CH-14) and 57.8 (CH-13) and further confirmed by the proton signals at δ_H_ 3.17 (1H, d, *J* = 3.6 Hz, H-14) and 3.25 (1H, d, *J* = 3.6 Hz, H-13). In addition, one methyl singlet, two methyl doublets, three aliphatic methine protons, four oxymethine protons, were observed in the ^1^H NMR spectrum of **5**.

**Table 4:** ^1^H (400 MHz, CDCl_3_) and ^13^C (100 MHz, CDCl_3_) NMR data and ^1^H–^1^H COSY and HMBC correlations for briarane **4**.

Position	δ_H_ (*J* in Hz)	δ_C_, Multiple	^1^H–^1^H COSY	HMBC
1		40.4, C		
2	4.20 br s	71.4, CH	H_2_-3	n. o. ^a^
3	2.45 br d (16.4); 2.13 m	37.4, CH_2_	H-2, H-4	n. o.
4	4.54 br d (5.2)	70.5, CH	H_2_-3, H_2_-16	n. o.
5		143.8, C		
6	5.06 br s	64.1, CH	H_2_-16	C-7
7	5.05 br s	77.5, CH		n. o.
8		83.8, C		
9	5.13 d (5.2)	73.2, CH	H-10	C-7, -8, -11, -17, acetate carbonyl
10	1.98 m	37.6, CH	H-9	n. o.
11	1.96 m	39.7, CH	H-12, H_3_-20	n. o.
12	4.58 d (4.4)	72.5, CH	H-11	C-1′
13	3.21 d (3.6)	57.3, CH	H-14	n. o.
14	3.09 d (3.6)	63.2, CH	H-13	C-13
15	1.16 s	16.6, CH_3_		C-1, -2, -10, -14
16	6.03 d (2.0); 6.02 br s	120.6, CH_2_	H-4, H-6	C-4, -5, -6
17	2.39 q (7.2)	44.1, CH	H_3_-18	C-8, -18, -19
18	1.14 d (7.2)	7.1, CH_3_	H-17	C-8, -17, -19
19		174.4, C		
20	1.06 d (7.2)	9.5, CH_3_	H-11	C-10, -11, -12
9-OAc		169.5, C		
	2.20 s	21.9, CH_3_		Acetate carbonyl
12-OC(O)CH_2_CH_2_CH_3_				
1′ 2′ 3′ 4′				
1′		172.7, C		
2′	2.35 t (7.2)	36.2, CH_2_	H_2_-3′	C-1′, -3′, -4′
3′	1.69 sext (7.2)	18.4, CH_2_	H_2_-2′, H_3_-4′	C-1′, -2′, -4′
4′	0.98 t (7.2)	13.7, CH_3_	H_2_-3′	C-2′, -3′
OH-8	3.35 s			C-8, -9

^a^ n. o. = not observed.

The gross structure of **5** was determined by 2D NMR studies. ^1^H NMR coupling information in the ^1^H–^1^H COSY spectrum of **5** enabled identification of the C-2/-3/-4, C-6/-7, C-9/-10/-11/-12, C-13/-14 and C-11/20 units. From these data and the HMBC correlations (Table 5), the connectivity from C-1 to C-14 and C-11 to C-20 could be established. One exocyclic double bond at C-5 was confirmed by the allylic coupling between H-4/H_2_-16 and H-6/H_2_-16 in the ^1^H–^1^H COSY spectrum and by the HMBC correlations between H_2_-16/C-4, -5, -6; H-4/C-16; and H-6/C-16. The ring junction C-15 methyl group was positioned at C-1 from the HMBC correlations between H-2/C-15, H-10/C-15, H-14/C-15, and H_3_-15/C-1, -2, -10, -14. Furthermore, the acetate esters positioned at C-6, C-9, and C-12 were established by the correlations between δ_H_ 5.73 (H-6), 5.26 (H-9), 4.63 (H-12) and the acetate carbonyls appearing at δ_C_ 170.1, 170.4, and 170.6, respectively. Thus, the remaining hydroxy group had to be positioned at C-8. These data, together with the HMBC correlations between H-9/C-17; H-17/C-8, -9, -18, -19; and H_3_-18/C-8, -17, -19, unambiguously established the molecular framework of **5**.

**Table 5:** ^1^H (400 MHz, CDCl_3_) and ^13^C (100 MHz, CDCl_3_) NMR data and ^1^H–^1^H COSY and HMBC correlations for briarane **5**.

Position	δ_H_ (*J* in Hz)	δ_C_, Multiple	^1^H–^1^H COSY	HMBC
1		41.4, C		
2	5.08 d (9.2)	71.9, CH	H-3	C-1, -3, -4, -14, -15
3	5.80 dd (12.0, 9.2)	134.7, CH	H-2, H-4	n. o. ^a^
4	6.07 d (12.0)	126.0, CH	H-3, H_2_-16	C-2, -3, -16
5		138.1, C		
6	5.73 d (9.6)	75.5, CH	H-7, H_2_-16	C-4, -5, -7, -16, acetate carbonyl
7	4.67 d (9.6)	81.6, CH	H-6	C-6
8		80.4, C		
9	5.26 d (7.6)	69.9, CH	H-10	C-7, -8, -10, -11, -17, acetate carbonyl
10	2.15 m	36.5, CH	H-9, H-11	C-1, -2, -8, -9, -11, -15, -20
11	2.04 m	37.3, CH	H-10, H-12, H_3_-20	C-12
12	4.63 d (4.4)	72.7, CH	H-11	C-10, -13, -14, -20, acetate carbonyl
13	3.25 d (3.6)	57.8, CH	H-13	n. o.
14	3.17 d (3.6)	62.6, CH	H-14	C-1, -10, -15
15	1.07 s	14.8, CH_3_		C-1, -2, -10, -14
16	5.63 s; 5.50 s	122.2, CH_2_	H-4, H-6	C-4, -5, -6
17	2.46 q (7.2)	45.0, CH	H_3_-18	C-8, -9, -18, -19
18	1.14 d (7.2)	6.2, CH_3_	H-17	C-8, -17, -19
19		175.1, C		
20	1.04 d (7.2)	9.3, CH_3_	H-11	C-10, -11, -12
6-OAc		170.1, C		
	2.09 s	21.3, CH_3_		Acetate carbonyl
9-OAc		170.4, C		
	2.18 s	21.9, CH_3_		Acetate carbonyl
12-OAc		170.6, C		
	2.13 s	21.1, CH_3_		Acetate carbonyl

^a^ n. o. = not observed.

**Figure 3:**
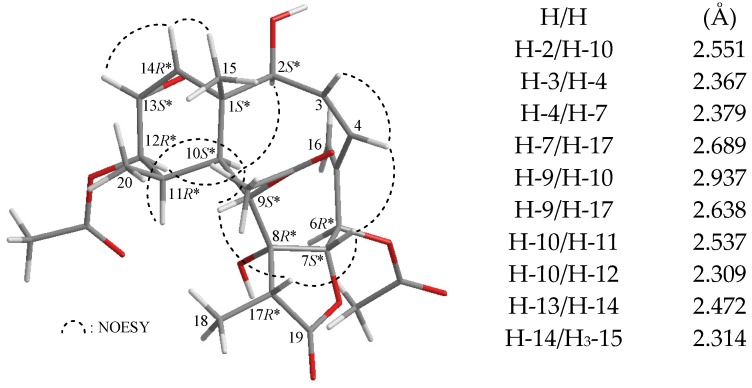
The computer-generated model of **5** using MM2 force field calculations and the calculated distances (Å) between selected protons with key NOESY correlations.

The relative stereochemistry of **5** was elucidated from the NOESY interactions observed in a NOESY experiment (Figure 3) and by the vicinal ^1^H–^1^H coupling constants analysis. In the NOESY experiment of **5**, H-10 gives correlations to H-2, H-9, H-11 and H-12, but not with H_3_-15 and H_3_-20, indicating that H-2, H-9, H-10, H-11, and H-12 are located on the same face of the molecule and assigned as α-protons, since C-15 and C-20 methyls are β-substituents at C-1 and C-11, respectively. The C-15 methyl protons were found to exhibit a response with H-14 and H-14 correlated with H-13 showing that the C-13/14 epoxy group was α-oriented. It was found that H-17 showed correlations with H-7 and H-9. Consideration of molecular models revealed that H-17 is reasonably close to H-7 and H-9 when H-17 and H-7 are β-oriented and H-9 and 8-hydroxy group are placed on the α face. H-7 showed a correlation with H-4, but not with H-6, and a large coupling constant (*J* = 9.2 Hz) was detected between H-6 and H-7, indicating that the dihedral angle between H-6 and H-7 is approximately 180°, and H-6 was α-oriented. The *cis* geometry of C-3/4 double bond was indicated by a correlation between H-3 (δ_H_ 5.80) and H-4 (δ 6.07) and confirmed by a 12.0 Hz coupling constant between these two olefin protons. Based on the consideration of a 3D model of **5**, and the chiral centers for briarane **5** are assigned as 1S*, 2S*, 6R*, 7S*, 8R*, 9S*, 10S*, 11R*, 12R*, 13S*, 14R*, and 17R*.

**Figure 4:**
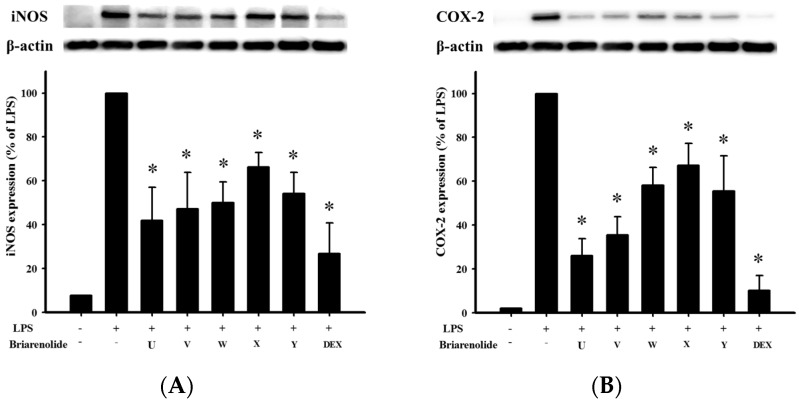
Effects of compounds briarenolides U–Y (**1**–**5**) on pro-inflammatory iNOS and COX-2 protein expression in the LPS-stimulated murine macrophage cell line RAW264.7. (**A**) The relative density of iNOS immunoblot; (**B**) the relative density of COX-2 immunoblot. The relative intensity of the LPS-stimulated group was taken to be 100%. Band intensities were quantified by densitometry and are indicated as the percent change relative to that of the LPS-stimulated group. Briarenolides U–Z (**1**–**5**) and dexamethasone (Dex) significantly inhibited LPS-induced iNOS and COX-2 protein expression in macrophages. The experiments were repeated three times (* *p* < 0.05, significantly different from the LPS-stimulated group).

In *in vitro* anti-inflammatory activity tests, the upregulation of the pro-inflammatory iNOS and COX-2 protein expression of LPS-stimulated RAW264.7 macrophage cells was evaluated using immunoblot analysis. At a concentration of 10 μM, briarenolides U–Y (**1**–**5**) were found to significantly reduce the levels of iNOS to 41.9%, 47.3%, 50.1%, 66.2%, and 54.3%, respectively, and these five compounds were also found to significantly reduce the levels of COX-2 to 26.1%, 35.6%, 58.1%, 67.2%, and 55.4%, respectively, relative to the control cells stimulated with LPS only (Figure 4).

**Table:** 

	iNOS	Cox-2
	Expression (% of LPS)	Expression (% of LPS)
Control	7.76 ± 1.99	2.2 ± 1.0
LPS	100 ± 0	100 ± 0
U (**1**)	41.9 ± 15.0	26.1 ± 7.7
V (**2**)	47.3 ± 16.5	35.6 ± 8.3
W (**3**)	50.1 ± 9.3	58.1 ± 8.1
X (**4**)	66.2 ± 6.7	67.2 ± 9.9
Y (**5**)	54.3 ± 9.6	55.4 ± 16.2
Dexamethasone ^a^	26.7 ± 14.1	10.1 ± 6.8

^a^ Dexamethasone (Dex) was used as a positive control.

## 3. Experimental Section

### 3.1. General Experimental Procedures

Melting points were determined on a Fargo apparatus (Panchum Scientific Corp. Kaohsiung, Taiwan) and are uncorrected. Optical rotation values were measured with a Jasco P-1010 digital polarimeter (Japan Spectroscopic Corporation, Tokyo, Japan). IR spectra were obtained on a Varian Digilab FTS 1000 FT-IR spectrophotometer (Varian Inc., Palo Alto, CA, USA); peaks are reported in cm^−1^. NMR spectra were recorded on a Varian Mercury Plus 400 NMR spectrometer (Varian Inc., Palo Alto, CA, USA) using the residual CHCl_3_ signal (δ_H_ 7.26 ppm) as the internal standard for ^1^H NMR and CDCl_3_ (δ_C_ 77.1 ppm) for ^13^C NMR. Coupling constants (*J*) are given in Hz. ESIMS and HRESIMS were recorded using a Bruker 7 Tesla solariX FTMS system (Bruker, Bremen, Germany). Column chromatography was performed on silica gel (230–400 mesh, Merck, Darmstadt, Germany). TLC was carried out on precoated Kieselgel 60 F_254_ (0.25 mm, Merck, Darmstadt, Germany); spots were visualized by spraying with 10% H_2_SO_4_ solution followed by heating. Normal-phase HPLC (NP-HPLC) was performed using a system comprised of a Hitachi L-7110 pump (Hitachi Ltd., Tokyo, Japan), a Hitachi L-7455 photodiode array detector (Hitachi Ltd., Tokyo, Japan), and a Rheodyne 7725 injection port (Rheodyne LLC, Rohnert Park, CA, USA). A semi-preparative normal-phase column (Hibar 250 × 10 mm, LiChrospher Si 60, 5 μm, Merck, Darmstadt, Germany) was used for HPLC. The reverse phase HPLC (RP-HPLC) was performed using a system comprised of a Hitachi L-7100 pump (Hitachi Ltd., Tokyo, Japan), a Hitachi L-2455 photodiode array detector (Hitachi Ltd., Tokyo, Japan), a Rheodyne 7725 injection port (Rheodyne LLC., Rohnert Park, CA, USA), and a Varian Polaris 5 C-18-A column (25 cm × 10 mm, 5 μm).

### 3.2. Animal Material

Specimens of the octocorals *Briareum* sp. were collected by hand using scuba equipment off the coast of southern Taiwan in July, 2011, and stored in a freezer (−20 °C) until extraction. The sample was extracted in August, 2011. A voucher specimen (NMMBA-TW-SC-2011-77) was deposited in the National Museum of Marine Biology & Aquarium. This organism was identified by comparison with previous descriptions [9,10,11,12,13].

### 3.3. Extraction and Isolation

Sliced bodies of *Briareum* sp. (wet weight, 6.32 kg; dry weight, 2.78 kg) were extracted with a mixture of methanol (MeOH) and dichloromethane (DCM) (1:1). The extract was partitioned between ethyl acetate (EtOAc) and H_2_O. The EtOAc layer was separated on silica gel and eluted using *n*-hexane/EtOAc (stepwise, 100:1, pure EtOAc) to yield 26 fractions, A–Z. Fractions M, N, O, and P were combined and further separated on silica gel and eluted using *n*-hexane/EtOAc (stepwise, 4:1, pure EtOAc) to afford 30 subfractions, M1–M30. Fraction M12 was further separated by silica gel and eluted using a mixture of DCM/MeOH (stepwise, 100:1–pure MeOH) to afford 26 subfractions M12A–M12Z. Fraction M12U was separated on reverse phase C18 column and eluted with MeOH and H_2_O (60:40) as the mobile phase to afford **5** (6.1 mg). Fraction V was chromatographed on silica gel and eluted using a mixture of DCM/EtOAc (stepwise, 20:1–pure EtOAc) to afford 14 subfractions, V1–V14. Fraction V8 was separated by NP-HPLC using a mixture of DCM/EtOAc (1:1) as the mobile phase to afford **3** (2.2 mg) and **4** (1.0 mg), respectively. Fraction V9 was separated by NP-HPLC using a mixture of DCM/EtOAc (1:1) to afford 25 subfractions V9A–V9Y. Fraction V9N was further repurified by RP-HPLC, using a mixture of MeOH/H_2_O (40:60) as the mobile phase to afford **1** (2.5 mg). Fraction V11 was separated by RP-HPLC using a mixture of MeOH/H_2_O (60:40) as the mobile phase to afford **2** (1.8 mg).

Briarenolide U (**1**): white powder; mp 311–312 °C (decomposed); [α]${}_{\text{D}}^{27}$ −13 (*c* 0.1, CHCl_3_); IR (neat) ν_max_ 3445, 1770, 1733 cm^−1^; ^1^H (400 MHz, CDCl_3_) and ^13^C (100 MHz, CDCl_3_) NMR data (see Table 1); ESIMS: *m*/*z* 535 [M + Na]^+^; HRESIMS: *m*/*z* 535.21480 (calcd. for C_25_H_36_O_11_ + Na, 535.21498).

Briarenolide V (**2**): white powder; mp 202–203 °C; [α]${}_{\text{D}}^{27}$ −16 (*c* 0.1, CHCl_3_); IR (neat) ν_max_ 3421, 1771, 1734 cm^−1^; ^1^H (400 MHz, CDCl_3_) and ^13^C (100 MHz, CDCl_3_) NMR data (see Table 2); ESIMS: *m*/*z* 531 [M + Na]^+^; HRESIMS: *m*/*z* 531.22025 (calcd. for C_26_H_36_O_10_ + Na, 531.22007).

Briarenolide W (**3**): white powder; mp 133–134 °C; [α]${}_{\text{D}}^{27}$ −25 (*c* 0.1, CHCl_3_); IR (neat) ν_max_ 3461, 1778, 1732 cm^−1^; ^1^H (400 MHz, CDCl_3_) and ^13^C (100 MHz, CDCl_3_) NMR data (see Table 3); ESIMS: *m*/*z* 539 [M + Na]^+^, 541 [M + 2 + Na]^+^; HRESIMS: *m*/*z* 539.16522 (calcd. for C_24_H_33_ClO_10_ + Na, 539.16545).

Briarenolide X (**4**): white powder; mp 180–181 °C; [α]${}_{\text{D}}^{27}$ −12 (*c* 0.1, CHCl_3_); IR (neat) ν_max_ 3461, 1780, 1732 cm^−1^; ^1^H (400 MHz, CDCl_3_) and ^13^C (100 MHz, CDCl_3_) NMR data (see Table 4); ESIMS: *m*/*z* 567 [M + Na]^+^, 569 [M + 2 + Na]^+^; HRESIMS: *m*/*z* 567.19687 (calcd. for C_26_H_37_ClO_10_ + Na, 567.19675).

Briarenolide Y (**5**): white powder; mp 196–197 °C; [α]${}_{\text{D}}^{27}$ −50 (*c* 0.3, CHCl_3_); IR (neat) ν_max_ 3445, 1770, 1732 cm^−1^; ^1^H (400 MHz, CDCl_3_) and ^13^C (100 MHz, CDCl_3_) NMR data (see Table 5); ESIMS: *m*/*z* 545 [M + Na]^+^; HRESIMS: *m*/*z* 545.19918 (calcd. for C_26_H_34_O_11_ + Na, 545.19933).

### 3.4. In Vitro Anti-Inflammatory Assay

The murine macrophage (RAW264.7) cell line was purchased from ATCC. The *in vitro* anti-inflammatory activity of Compounds **1**−**5** was measured by examining the inhibition of lipopolysaccharide (LPS)-induced upregulation of pro-inflammatory iNOS (inducible nitric oxide synthase) and COX-2 (cyclooxygenase-2) protein expression in macrophage cells using Western blotting analysis [14,15,16]. Briefly, inflammation in macrophages was induced by incubating them for 16 h in a medium containing only LPS (10 ng/mL) without compounds. For the anti-inflammatory activity assay, Compounds **1**−**5** and dexamethasone (10 μM) were added to the cells 10 min before the LPS challenge. The cells were lysed then for western blot analysis. The immunoreactivity data were calculated with respect to the average optical density of the corresponding LPS-stimulated group. For statistical analysis, the data were analyzed by a one-way analysis of variance (ANOVA), followed by the Student–Newman–Keuls *post hoc* test for multiple comparisons. A significant difference was defined as a *p*-value of <0.05.

## 4. Conclusions

Our continuing investigations demonstrated that the octocorals belonging to the genus *Briareum* are good sources of briarane-type natural products. Briarenolides U−Y (**1**−**5**) are potentially anti-inflammatory and may become lead compounds in future marine anti-inflammation drug development [17,18]. These results suggest that continuing investigation of new briaranes together with the potentially useful bioactivities from this marine organism are worthwhile for future drug development. The octocoral *Briareum* sp. had been transplanted to culturing tanks located in the National Museum of Marine Biology & Aquarium, Taiwan, for extraction of additional natural products to establish a stable supply of bioactive material.
